# Supplementary material for: Stress-Induced Premature Senescence Related to Oxidative Stress in the Developmental Programming of Nonalcoholic Fatty Liver Disease in a Rat Model of Intrauterine Growth Restriction
Source: Antioxidants (Basel). 2022 Aug 29;11(9):1695. doi: 10.3390/antiox11091695 (PMC9495674; doi:10.3390/antiox11091695)
Supplement: Supplementary file 1 [file antioxidants-11-01695-s001.zip › Supplementary figures.pdf]

Stress-induced premature senescence related to oxidative stress in the developmental programming of nonalcoholic fatty liver disease in a rat model of intrauterine growth restriction

Basile Keshavjee, Valentine Lambelet, Hanna Coppola, David Viertl, John O. Prior, Laurent Kappeler, Jean-Baptiste Armengaud, Jean-Pierre Chouraqui, Hassib Chehade, Paul-Emmanuel Vanderriele, Manon Allouche, Anne Balsiger, Alexandre Sarre, Anne-Christine Peyter, Umberto Simeoni and Catherine Yzydorczyk

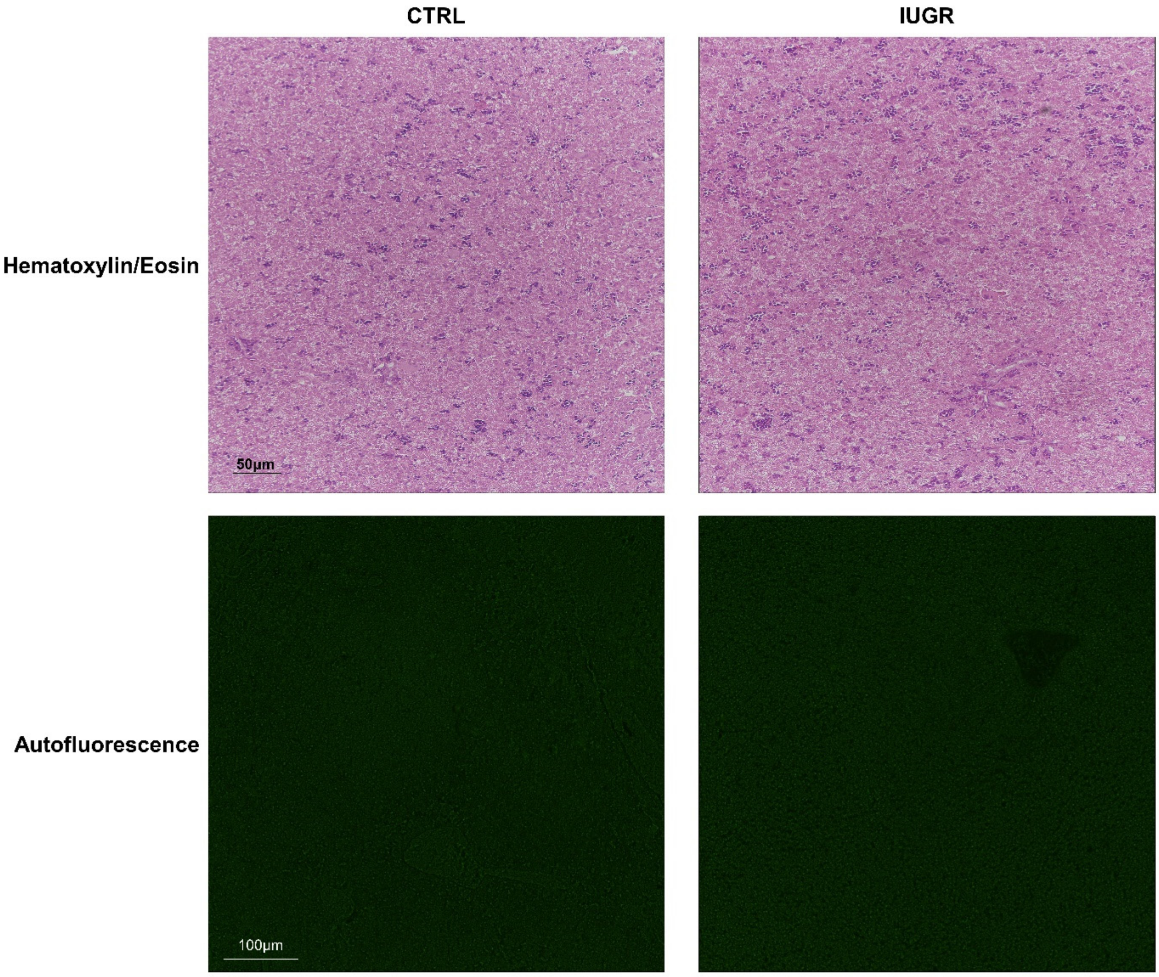

Supplementary Figure S1. Liver structure and lipofuscin detection in CTRL and IUGR males at birth.
The liver structure was evaluated using hematoxylin/eosin (H/E) staining and the lipofuscin detection was investigated using autofluorescence. n = 4 animals/group. Magnification (20×). Scale bar = 50 and 100 µm.

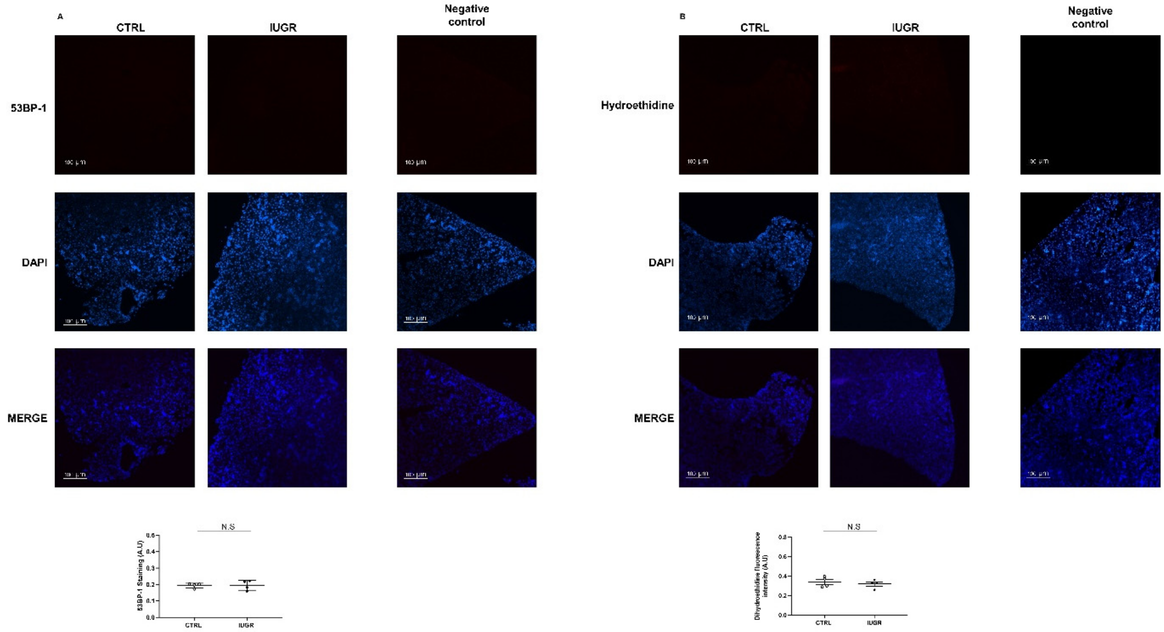

Supplementary Figure S2. Oxidative stress evaluation in the liver from CTRL and IUGR males at birth. The DNA double-strand breaks were evaluated by 53BP-1 staining (**A**) and the superoxide anion production was evaluated using hydroethidine staining (**B**). A negative control was performed. Nuclei were counterstained with DAPI. N.S: not significant. n = 4 animals/group. Magnification (20×). Scale bar = 100 µm.
